# Prescribing Trends of Mood Stabilisers Within an Assertive Outreach Team (AOT) in York

**DOI:** 10.1192/bjo.2026.11858

**Published:** 2026-06-30

**Authors:** Christiana Elisha-Aboh, Edward Marson, Thomas Riley, Christiana Akubuiro

**Affiliations:** Tees, Esk and Wear Valley Foundation Trust, York, United Kingdom

## Abstract

**Aims:**

**Background:**

Lithium and Valproate remain established mood stabilisers with strong evidence for efficacy in bipolar disorder and affective instability. Contemporary clinical practice increasingly reflects a shift towards alternatives such as lamotrigine and atypical antipsychotics. This shift appears driven not only by clinical considerations but also by regulatory, safety and service-level factors. Valproate prescribing has declined markedly among women of childbearing age due to teratogenic risks and regulatory restrictions, while lithium use is challenged by monitoring requirements and systemic barriers within services. These influences may contribute to the underutilisation of effective treatments, raising concerns about the impact of administrative burden, risk management, and evolving prescribing culture on real-world pharmacological practice.

Aim: This study aimed to examine prescribing patterns of lithium, valproate, and alternative mood stabilisers within an AOT cohort across two time points six months apart. It sought to quantify changes in the use of lithium, valproate, and lamotrigine and to explore whether observed trends reflect broader clinical, regulatory, and service-related influences on prescribing practice.

**Methods:**

A retrospective observational review of clinical records was conducted including all patients under the care of the AOT at two defined points, six months apart. Patients prescribed mood stabilisers were identified. Data collected included current mood stabiliser (lithium, valproate, lamotrigine, or other), demographic characteristics (age and sex), clinical indication, and changes in prescribing between time points. Descriptive statistics were used to calculate the proportion of patients receiving each mood stabiliser at both time points. Changes in prescribing patterns over the six-month period were analysed and compared across medication groups to identify emerging trends.

**Results:**

The AOT caseload increased from 73 patients in July 2025 to 85 patients in January 2026. Lithium prescribing decreased slightly from 6 patients (8.2%) to 5 patients (5.9%), while valproate use remained numerically stable at 5 patients (6.8% vs 5.9%). Lamotrigine prescribing increased from 3 patients (4.1%) to 6 patients (7.1%), representing the most notable change over the study period. Carbamazepine use declined from 1 patient (1.4%) to none. Overall, lithium and valproate use remained stable or declined slightly, while lamotrigine use increased despite changes in caseload and patient turnover, indicating a gradual shift in mood stabiliser prescribing patterns within the AOT population.

**Conclusion:**

Findings mirror wider trends in psychiatric prescribing, characterised by a relative decline in lithium use due to monitoring and systemic barriers, regulatory driven constraints on valproate, and increasing reliance on lamotrigine and alternative agents. These results suggest that effective mood stabilisers may be underutilised not because of reduced efficacy but due to evolving regulatory frameworks, risk management priorities, and service-level pressures. Understanding these influences is important for informing prescribing practice and ensuring that clinical decision-making remains balanced between safety, feasibility, and therapeutic effectiveness in complex mental health populations.

